# Association of Concussion With an Increased Risk of Psychotic Disorders: A Retrospective Cohort Study

**DOI:** 10.7759/cureus.114341

**Published:** 2026-08-11

**Authors:** Srishti Reddy, Thayjas A Patil, Eduardo D Espiridion

**Affiliations:** 1 Obstetrics and Gynecology, Drexel University College of Medicine, Reading, USA; 2 Psychiatry, Drexel University College of Medicine, Philadelphia, USA

**Keywords:** concussion, head trauma adults, mild head injury, post-concussive syndrome, psychosis, psychotic disorder, trauma

## Abstract

Background: Head trauma has a well-documented association with increased incidence of psychiatric disorders, especially depression, anxiety, and post-traumatic stress disorder (PTSD). However, the relationship between traumatic brain injury (TBI) and subsequent psychotic disorders remains poorly characterized. Despite their relative rarity, psychotic disorders are highly debilitating, making clarification of this association clinically important, given the substantial public health burden of mild traumatic brain injury (mTBI). Whether this risk reflects mechanisms specific to head injury or trauma exposure more broadly remains unclear.

Methods: Using data from 3,023,268 propensity score-matched patients from 68 responding providers in the TriNetX US Collaborative Network, this retrospective cohort study compared patients with mTBI, defined by concussion or postconcussional syndrome (PCS), with patients with non-head trauma. Patients with psychotic outcomes before the index event were excluded. Outcomes included schizophrenia, schizotypal disorder, and brief psychotic disorder.

Results: Over a 20-year follow-up period, 7,681 patients in the mTBI cohort and 3,178 patients in the non-head trauma cohort developed a psychotic disorder, corresponding to an absolute risk of 0.51% in the mTBI cohort and 0.21% in the non-head trauma cohort. mTBI was associated with a higher risk of psychotic disorder compared with non-head trauma (Risk Ratio (RR) 2.42, 95% CI 2.33-2.53, Odds Ratio (OR) 2.43, 95% CI 2.33-2.53). Kaplan-Meier analysis showed lower psychosis-free survival probability in the mTBI cohort at the end of follow-up (97.31% vs 98.83%), and Cox regression showed a significantly increased hazard of psychotic disorder (Hazard Ratio (HR) 2.125, 95% CI 2.04-2.22, p<0.001).

Conclusions: Although new-onset psychotic disorders were rare in both cohorts, patients with mTBI were more than twice as likely to develop psychotic disorders as patients with non-head trauma. These findings support an association between concussion/PCS and later psychotic illness and highlight the need for further work on mechanisms, risk modifiers, and early identification strategies.

## Introduction

Approximately 3 million patients seek medical attention for traumatic brain injury (TBI) annually in the United States [1]. On a global scale, data from 2019 estimate an annual burden of 27.16 million new cases of TBI alone and a prevalence of 48.99 million cases [2]. Mild TBI (mTBI), commonly referred to as concussion, accounts for up to 90% of all TBI cases [3]. Consequently, mTBI represents a large global population that is potentially at risk of a range of long-term neuropsychiatric and medical sequelae that continue to be elucidated [4, 5].

Distinguishing mTBI from moderate and severe TBI is critical as these conditions vary greatly in pathophysiology, acute management, and long-term outcomes [3, 6]. Although the definition of mTBI remains somewhat controversial, current consensus supports the definition outlined by the American Congress of Rehabilitation Medicine (ACRM) as revised by the WHO Collaborating Centre Task Force on Mild Traumatic Brain Injury [3]. The ACRM defines mTBI as head trauma resulting in transient alterations in consciousness, memory, or neurologic function, a Glasgow Coma Scale score of 13-15, and the absence of features or duration of symptoms consistent with moderate or severe TBI criteria. While concussion is often used interchangeably with mTBI, it is specifically used for mTBI when there is no indication for imaging or neuroimaging is normal [3].

Within the field of psychiatry, the link between head trauma and psychiatric sequelae is well established when contrasted to the general population [1, 7-13]. Studies thus far have strongly supported depression, anxiety, and post-traumatic stress disorder (PTSD) as the most likely psychiatric sequelae of TBI [7-13]. Additionally, one meta-analysis of nine case-controlled population-based studies demonstrated 1.65 times the odds of developing schizophrenia in patients with a history of TBI as opposed to those without (95% CI = 1.17-2.32) [13]. Despite their relative rarity, psychotic disorders are associated with profound functional impairment and comorbidity [14]. Further, most prior studies have compared patients with TBI to the general population, making it difficult to distinguish the effects of head trauma from the known association with trauma exposure more broadly [15]. Whether the increased risk of psychotic disorders persists when compared with individuals exposed to non-head trauma remains unclear.

Concussions and mTBI are often accompanied by post-concussion symptoms, ranging from physical symptoms to cognitive, emotional, and behavioral deficits. Although symptoms typically resolve within days to weeks of the injury, a subset of approximately 30% of patients experience recurrent or persistent symptoms consistent with post-concussion syndrome (PCS) [1, 3]. Because most psychiatric sequelae occur within the first six months of injury, current guidelines recommend psychiatric screening during this period, with additional follow-up guided by clinical judgement and patient-specific risk factors [16, 17].

Increasing evidence encourages conceptualizing TBI as a chronic condition rather than an acute condition with full resolution of subsequent symptoms [4, 5]. Long-term studies, including the TRACK-TBI LONG Study, have demonstrated that functional and psychiatric outcomes following TBI continue to evolve even years after injury [4]. However, like many existing studies, TRACK-TBI also excluded psychotic disorders from psychiatric outcome measures and focused on typical anxiety and depressive symptoms. Consequently, the long-term risk for psychotic sequelae following mTBI remains poorly understood.

Given the substantial burden of mTBI and the disabling nature of psychotic disorders, further characterization of this association may have important implications for long-term management. Therefore, we designed a retrospective cohort study of patients with mTBI, including concussion and PCS, using patients with non-head trauma as controls. The primary objective of this study was to evaluate whether mTBI is associated with a higher incidence of new-onset psychotic disorders, defined as a composite outcome comprising schizophrenia, schizotypal disorder, and brief psychotic disorder, over a 20-year follow-up period, compared with non-head trauma. As a secondary analysis, we additionally examined the total number of psychotic disorder diagnoses per patient to characterize the overall burden of diagnoses across cohorts.

## Materials and methods

Study design

This retrospective observational cohort study evaluated differences in the incidence of psychotic disorders among patients with mTBI compared with patients who experienced non-head trauma, using data obtained from the TriNetX global health research network. TriNetX grants access to EPIC-based electronic health record data from major healthcare organizations (HCOs). The study included data from 69 HCOs participating in the US Collaborative Network. This study was exempt from informed consent because it was based on secondary analysis of existing de-identified data and involved neither intervention nor direct interaction with human participants, consistent with the de-identification standard defined in Section §164.514(a) of the HIPAA Privacy Rule [18]. TriNetX holds ISO 27001:2022 certification and operates an Information Security Management System (ISMS) to safeguard data security. The version of TriNetX available in September 2025 was used for this analysis.

Cohort selection and matching

The analysis included two matched cohorts: Cohort 1 (n = 1,511,634), representing patients with mTBI, and Cohort 2 (n = 1,511,634), representing patients with non-head trauma. Propensity scores were generated using logistic regression within the TriNetX platform, with cohort assignment (mTBI vs. non-head trauma) as the dependent variable and the matching covariates as predictors. Patients were matched 1:1 using greedy nearest-neighbor matching without replacement, to balance the cohorts with respect to four demographic variables: current age, age at index event, and sex (female and male). These variables were selected because they represent core demographic covariates consistently available and reliably coded across all participating HCOs. Other potential confounders, including prior psychiatric diagnoses, such as mood disorders, substance use disorders, PTSD, family history of psychosis, or socioeconomic factors, were not able to be included due to constraints imposed by the TriNetX platform, which limits the number of variables that can be simultaneously matched as cohort size increases.

Inclusion and exclusion criteria

The query criteria for Cohort 1, mTBI, required inclusion of “concussion” (ICD10CM:S06.0) or “postconcussional syndrome” (ICD10CM:F07.81). Query criteria for Cohort 2, non-head trauma, required inclusion of “injury, poisoning, and certain other consequences of external causes” (ICD10CM:S00-T88) and exclusion of “injuries to the head” (ICD10CM:S00-S09), “Concussion” (ICD10CM:S06.0), “postconcussional syndrome” (ICD10CM:F07.81), and “poison by, adverse effects of and underdosing of drugs, medicaments and biological substances” (ICD10CM:T36-T50). Patients in both cohorts were identified on the basis of these query definitions, with the index event occurring at any point during the preceding 20 years. Individuals with a documented outcome before the index event were excluded from the analysis. No other psychiatric diagnoses were used as exclusion criteria, such as mood disorders, substance use disorders, PTSD, or family history of psychosis.

Outcome measures

Outcomes of interest included “schizophrenia” (ICD10CM:F20), “schizotypal disorder” (ICD10CM:F21), and “brief psychotic disorder” (ICD10CM:F23).

Statistical analysis

Baseline cohort characteristics were analyzed using an independent two-sample t-test for continuous variables and a chi-squared test for categorical variables, with unequal variances assumed. In addition to binary outcome analyses, an independent two-sample t-test was used to compare the mean number of psychotic disorder diagnosis instances per patient between cohorts, treating the total count of qualifying diagnoses per individual as a continuous variable. This analysis was conducted to characterize the overall burden of psychotic disorder diagnoses across cohorts and complements the primary binary outcome analyses. The distribution of categorical variables was compared across cohorts, and each subgroup was evaluated against all remaining patients within the same broader category. Associations between cohort membership and each outcome were assessed by comparing the proportion of patients who developed the selected outcomes. The principal measures of association included risk difference, risk ratio, and odds ratio. Time-to-event analyses were performed with Kaplan-Meier methods to estimate outcome probabilities over time, with follow-up beginning at the index event (date of qualifying mTBI or non-head trauma diagnosis) and continuing until outcome occurrence, loss to follow-up, or the end of the 20-year study window, whichever came first. Patients were censored at their last recorded encounter within the TriNetX network if they left the cohort during follow-up without experiencing the outcome. Reported measures included median survival (defined as the time in days until survival fell below 50%), survival probability at the end of the time window, the log-rank test, and the hazard ratio. The proportionality assumption for the Cox regression model was evaluated using TriNetX's built-in proportionality test, reported alongside the hazard ratio. For statistical analyses, TriNetX uses several programming environments and software packages, including Apache Commons Math 3.6.1 (Apache Software Foundation, Wilmington, DE, USA), Hmisc 4.4-1 and Survival 3.2-3 (R Foundation for Statistical Computing, Vienna, Austria), Lifelines 0.22.4 (Cameron Davidson-Pilon, Toronto, ON, Canada), Matplotlib 3.5.1 (Matplotlib Development Team), NumPy 1.21.5 (NumPy Developers), pandas 1.3.5 (The pandas Development Team), SciPy 1.7.3 (SciPy Developers), and statsmodels 0.13.2 (Statsmodels Development Team), implemented in Java 11.0.16 (Oracle Corporation, Austin, TX, USA), R 4.0.2 (R Foundation for Statistical Computing, Vienna, Austria), and Python 3.7 (Python Software Foundation, Beaverton, OR, USA).

## Results

Of the available 69 HCOs in the US Collaborative Network, a total of 68 providers responded with available patient data. Before propensity score matching, Cohort 1 had 1,554,257 patients match the query criteria for mTBI, and Cohort 2 had 22,618,203 patients match the query criteria for non-head trauma. Cohort characteristics before and after propensity score matching can be seen in Table 1 and Table 2, respectively.

**Table 1:** Cohort characteristic before propensity score matching Continuous variables were compared using an independent two-sample t-test, and categorical variables were compared using chi-square tests. Standardized differences are also reported as a measure of baseline imbalance between cohorts. Bold p values designate statistically significant results using an α<0.05. PCS: postconcussional syndrome

Cohort characteristics before propensity matching	Cohort 1 (concussion/PCS)	Cohort 2 (non-head trauma)	p value
Total patients	1,554,257	22,618,203	
Current age	38.4 +/- 21.3	45.5 +/- 23.7	<0.001
Age at index	31.5 +/- 21.3	38.5 +/- 23.8	<0.001
Gender			
Female	745,725	10,644,444	0.091
Male	726,607	10,420,345	<0.001
Ethnicity			
Not Hispanic or Latino	1,036,840	13,827,106	<0.001
Unknown ethnicity	323,843	5,335,003	<0.001
Hispanic or Latino	150,951	2,838,848	<0.001
Race			
White	1,001,318	13,632,887	<0.001
Black or African American	204,989	3,181,333	<0.001
Unknown race	156,449	2,359,042	<0.001
Other race	81,619	1,306,376	<0.001
Asian	44,457	791,449	<0.001
Native Hawaiian or other Pacific Islander	14,890	171,299	<0.001
American Indian or Alaska Native	7,921	103,571	<0.001

**Table 2:** Cohort characteristics after propensity score matching Continuous variables were compared using an independent two-sample t-test, and categorical variables were compared using chi-square tests. Standardized differences are also reported as a measure of baseline imbalance between cohorts. Bold p values designate statistically significant results using an α<0.05. PCS: postconcussional syndrome

Cohort characteristics after propensity matching	Cohort 1 (concussion/PCS)	Cohort 2 (non-head trauma)	p value
Total patients	1,511,634	1,511,634	
Current age	38.4 +/- 21.3	38.4 +/- 21.3	0.998
Age at index	31.5 +/- 21.3	31.5 +/- 21.3	0.999
Gender			
Female	745,725	745,730	0.995
Male	726,607	726,607	1.000
Ethnicity			
Not Hispanic or Latino	1,036,840	951,573	<0.001
Unknown ethnicity	323,843	375,388	<0.001
Hispanic or Latino	150,951	184,673	<0.001
Race			
White	1,001,318	926,789	<0.001
Black or African American	204,989	234,519	<0.001
Unknown race	156,449	177,907	<0.001
Other race	81,619	98,992	<0.001
Asian	44,457	54,050	<0.001
Native Hawaiian or other Pacific Islander	14,890	11,919	<0.001
American Indian or Alaska native	7,912	7,458	<0.001

Analysis before propensity score matching revealed significant differences in current age (p<0.001) and age at index (p<0.001), as seen in Table 1. Significant differences were also observed in male gender (p<0.001), but not female gender (p=0.091). Additionally, individual race and ethnicity categories, including “Not Hispanic or Latino”, “Unknown Ethnicity “, “Hispanic or Latino”, “White”, “Black or African American”, “Unknown Race”, “Other Race”, “Asian”, “Native Hawaiian or Other Pacific Islander”, “American Indian or Alaska Native” were all significantly different (p<0.001).

Cohort characteristics analysis after propensity score matching for current age, age at index, female gender, and male gender, outlined in Table 2, revealed no significant differences between cohorts in current age (p=0.998), age at index (p=0.999), female gender (p=0.995), and male gender (p=1.000). The individual race and ethnicity categories were still significantly different between cohorts after propensity score matching (p<0.001). The resulting matched cohorts included 1,511,634 patients in Cohort 1, mTBI, and 1,511,634 patients in Cohort 2, non-head trauma.

Over a follow-up period of 20 years, our data showed that 7,681 patients in the mTBI cohort and 3,178 patients in the non-head trauma cohort developed at least one of the specified psychiatric outcomes (Table 3). Outcome results and subsequent analyses can be seen in Tables 3-9.

**Table 3:** Patient outcomes by cohort PCS: postconcussional syndrome

Cohort	Patients in cohort	Patients excluded because of outcome prior to time window	Patients with outcome	Risk
1. (Concussion/PCS)	1,503,548	8,086	7,681	0.00511
2. (Non-head trauma)	1,508,039	3,595	3,178	0.00211

**Table 4:** Risk difference, risk ratio, and odds ratio analysis CI: confidence interval; z: Z-score; bold p values designate statistically significant results using an α<0.05.

Analysis	Value	95% CI	z	p value
Risk difference	0.003	(0.003, 0.003)	43.446	<0.001
Risk ratio	2.424	(2.326, 2.526)	N/A	N/A
Odds ratio	2.431	(2.333, 2.534)	N/A	N/A

**Table 5:** Descriptive statistics Descriptive statistics reflect the distribution of total psychotic disorder diagnosis counts per patient (the cumulative number of qualifying diagnosis instances per individual across the follow-up period). Mean and standard deviation are calculated across all patients in each cohort, including those with zero qualifying diagnoses. “Median (1+ instances)" refers to the median count among patients who received at least one qualifying diagnosis. PCS: postconcussional syndrome

Cohort	Patients in cohort		Patients with outcome	Mean		Standard deviation	Median		Median (1+ instances)
1. (Concussion/PCS)	1,503,548		7,681	0.025		1.060	0		2
2. (Non-head trauma)	1,508,039		3,178	0.009		0.830	0		1

**Table 6:** Two-sample t-test Results are from an independent two-sample t-test. t: t-test statistic; df; degrees of freedom. Bold p values indicate statistical significance at α<0.05.

t	df	p value
14.436	3011585	<0.001

**Table 7:** Kaplan-Meier Survival analysis PCS: postconcussional syndrome

Cohort	Patients in cohort	Patients with outcome	Median survival (days)	Survival probability at end of time window
1. (Concussion/PCS)	1,503,548	7,681	N/A	97.31%
2. (Non-head trauma)	1,508,039	3,178	N/A	98.83%

**Table 8:** Log-rank test Results are from a log-rank test comparing Kaplan-Meier survival curves. χ²: chi-square test statistic; df: degrees of freedom. Bold p values indicate statistical significance at α<0.05.

Test	χ^2^	df	p value
Log-rank test of outcome	1338.723	1	<0.001

**Table 9:** Hazard ratio analysis CI: confidence interval; χ^2^: Chi-Square; df: degrees of freedom; bold p values designate statistically significant results using an α<0.05.

Hazard ratio	95% CI	χ^2^	df	p value
2.125	(2.039, 2.215)	14.739	1	<0.001

The risk ratio revealed how likely exposure to mTBI versus non-head trauma was to predict the development of a psychotic disorder. Relative risk analysis showed that patients with a history of concussion or PCS had a 2.424-fold higher risk of developing a psychotic disorder compared with non-head trauma patients (RR = 2.424, 95% CI 2.33-2.53) (Table 4). Further analysis of the odds ratio showed 2.431 times higher odds of developing a psychotic disorder in the mTBI group compared to controls (OR = 2.431, 95% CI 2.33-2.53) (Table 4).

The primary outcome in this study was defined as the binary occurrence of any qualifying psychotic disorder diagnosis (schizophrenia, schizotypal disorder, or brief psychotic disorder) during the follow-up period. Table 5 and Table 6, however, analyze the total count of qualifying psychotic disorder diagnosis instances per patient (the cumulative recorded diagnoses per individual across follow-up); a distinct but complementary analysis intended to characterize the burden of psychotic disorder diagnoses across cohorts. Despite a significant odds ratio for the binary outcome, the median number of diagnosis instances was 0 in both cohorts, indicating that most patients never received any qualifying psychotic disorder diagnosis during follow-up (Table 5). Among patients who did develop a qualifying psychotic disorder (those with at least one recorded instance), the median count was 2 in the mTBI cohort and 1 in the non-head trauma cohort. Descriptive statistics further revealed a higher mean count and greater variability in diagnosis instances among the mTBI patients (mean = 0.025, SD = 1.06) compared with non-head trauma patients (mean = 0.009, SD = 0.83). The high standard deviation relative to the mean in both groups is consistent with a right-skewed count distribution, in which most patients contributed zero events, while a smaller subset accumulated multiple qualifying diagnoses. This pattern may reflect heterogeneity in risk and clinical trajectory among subgroups of patients with mTBI.

An independent two-sample t-test comparing mean diagnosis counts between cohorts was highly significant (t = 14.436, df = 3,011,585, p<0.001) (Table 6), confirming that the difference in mean diagnosis burden was not attributable to chance. Given the large sample size, this test provides statistical support for a difference in the overall burden of psychotic disorder diagnoses between cohorts, complementing the binary outcome analyses described above.

To better understand the temporality of outcome measure development over the course of the study period, survival analysis, log-rank test, and hazard ratio analysis were conducted. The Kaplan-Meier survival curve estimates the probability of remaining free from psychotic disorder diagnosis by the end of the 20-year follow-up period. At the end of follow-up, survival probability was 97.31% for the mTBI cohort and 98.83% for the non-head trauma cohort (Table 7). A log-rank test provided validity to a difference between cohort survival rates (Table 8). Cox regression additionally resulted in a hazard ratio of 2.125 (95% CI 2.04-2.22, p<0.001), indicating that at any given time individuals with a history of mTBI were 2.125 times more likely to develop any one of the psychotic disorders compared to those with non-head trauma (Table 9).

## Discussion

Our study demonstrated that patients with mTBI or PCS had significantly greater odds of developing a psychotic disorder than patients with non-head trauma, observed up to 20 years after the index event, though the absolute risk remained low in both groups (0.51% vs. 0.21%). Survival analysis similarly demonstrated a lower probability of remaining free of psychotic disorders, with an over two-fold increased risk of developing one of the given disorders at any given time. These results align with the hypothesis that mTBI is associated with an increased risk for psychotic sequelae beyond that associated with trauma exposure alone.

Our results are consistent with previous studies demonstrating an increased risk of psychotic disorders following mTBI. Meta-analysis of both pediatric and adult TBI demonstrated elevated odds of psychotic symptoms or diagnoses (1.80 (95% CI 1.11-2.95) in pediatric populations and 1.65 (95% CI = 1.17-2.32) in adult populations) [10, 13]. Family studies demonstrate that TBI had a markedly larger influence on the incidence of schizophrenia in those with a family history of schizophrenia, with a pooled odds ratio of 2.8 (95% CI =1.76-4.47) [13, 19]. Unlike most prior investigations that used the general population as controls, our study employed patients with non-head trauma as a comparison cohort, suggesting that the observed association may reflect mechanisms specific to head injury rather than trauma exposure more broadly.

The pathophysiology of psychotic disorders remains incompletely understood. Current consensus emphasizes neurobiological mechanisms due to dysregulation in glutamatergic and dopaminergic pathways in conjunction with trauma-induced cognitive-affective changes. PTSD is theorized to play a mediating role in trauma-related psychosis and has been associated with increased odds of psychotic disorders, particularly amongst adolescents [20]. In the context of head trauma, growing evidence supports the role of neuroinflammation in the pathogenesis of psychiatric illness following TBI. Central and peripheral inflammatory markers, including microglial activation and cytokine dysregulation, have been implicated as potential contributors to psychiatric sequelae after mTBI [21, 22]. Additionally, a meta-analysis of placebo-controlled randomized clinical trials has demonstrated potentially favorable neurological and functional outcomes with the use of anti-inflammatory medications after TBI [23]. Although studies thus far have focused on PTSD and depression as psychiatric sequelae of TBI, our findings warrant further investigation into inflammatory mechanisms and their potential role in the development of psychotic disorders following mTBI.

In conjunction with previous research, our findings suggest that psychotic disorders are an important neuropsychiatric outcome following concussion, with risk extending well beyond the acute and subacute phases of injury. The observed association over a 20-year period supports the emerging view of mTBI as a chronic condition with evolving outcomes and highlights the importance of continued investigation into long-term psychiatric monitoring, underlying mechanisms, and strategies aimed at mitigating neuropsychiatric sequelae.

Although TriNetX offers access to a large multicenter dataset, several limitations should be considered when interpreting these findings. The platform draws from EPIC-based medical records dating back to 2006, and temporary nonparticipation by some HCOs may result in small shifts in cohort size and baseline characteristics depending on when the query is performed, which may reduce reproducibility. Baseline differences between cohorts remained significant, and only four variables could be balanced through propensity score matching because of platform constraints. As a result, the extent to which residual differences between cohorts influenced the observed outcomes could not be fully determined, particularly in the absence of patient-level raw data. Even so, cohort identification was conducted using the same automated query process and the same predefined inclusion and exclusion rules for each group. In addition, because the data were derived from large U.S. healthcare organizations, the findings may not be fully generalizable to smaller practice settings or to populations outside the United States. Furthermore, because cohort and outcome classification relied on International Classification of Diseases, Tenth Revision, Clinical Modification (ICD-10-CM) [24] diagnostic codes rather than confirmed clinical evaluation, this study is subject to potential information biases such as coding inaccuracies, variability in diagnostic practices across providers, and underreporting, which could result in misclassification of exposure or outcome status. The inability to access raw patient-level data, including information on attrition, comorbid conditions, family history, and socioeconomic factors, further limited evaluation of potential confounders that may have influenced the results. In particular, prior diagnoses of mood disorders, substance use disorders, and PTSD, as well as family history of psychosis, were not available for matching or exclusion and represent important unmeasured confounders. Additionally, patients with concussion or PCS may have had a differing frequency of healthcare encounters than the comparison cohort, raising the possibility that the observed association partly reflects surveillance and ascertainment bias rather than a true difference in underlying risk. It was also not possible to further stratify the severity of mTBI or non-head trauma within the available dataset, as the incidence of psychotic outcomes may vary according to injury severity. Despite these constraints, the study provides meaningful information regarding the association between trauma exposure and psychotic outcomes across the analyzed cohorts.

## Conclusions

This study aimed to evaluate the difference in incidence of psychotic disorders in patients who have experienced mTBI compared to patients who have experienced non-head trauma. The results showed that although the absolute risk of developing a psychotic disorder remained low in both cohorts (0.51% vs. 0.21%), mTBI was associated with more than twice the likelihood of developing a new-onset psychotic disorder compared to patients with non-head trauma. Cox hazard ratio analysis similarly showed an association between mTBI and a twofold likelihood of developing psychotic disorders at any given time. Given the study’s limitations, such as lack of complete propensity score matching, control over all potential confounders, or ability to differentiate trauma severity more closely, further research is needed on the psychotic sequelae of mTBI and non-head trauma. Given recent evidence linking inflammation with depression and PTSD after mTBI, it is reasonable that future studies aimed at understanding treatment of psychotic sequelae consider this pathogenic mechanism. Continued research will need to further promote early intervention and systematic reforms for often preventable forms of mild head injury and trauma, given its significant association with future morbidity.
